# Polymeric Mixed Micelle-Loaded Hydrogel for the Ocular Delivery of Fexofenadine for Treating Allergic Conjunctivitis

**DOI:** 10.3390/polym16162240

**Published:** 2024-08-07

**Authors:** Sherouk A. El-Shahed, Doaa H. Hassan, Mohamed A. El-Nabarawi, Doaa Ahmed El-Setouhy, Menna M. Abdellatif

**Affiliations:** 1 Department of Pharmaceutics, College of Pharmaceutical Sciences and Drug Manufacturing, Misr University for Science and Technology, Giza 12566, Egypt; sherouk.elshahed@must.edu.eg (S.A.E.-S.); doaa.hassan@must.edu.eg (D.H.H.); 2 Department of Pharmaceutics and Industrial Pharmacy, Faculty of Pharmacy, Cairo University El-Kasr El-Aini Street, Cairo 11562, Egypt; mohamed.elnabarawi@pharma.cu.edu.eg (M.A.E.-N.); doaa.elsetouhy@pharma.cu.edu.eg (D.A.E.-S.); 3 Department of Industrial Pharmacy, College of Pharmaceutical Sciences and Drug Manufacturing, Misr University for Science and Technology, Giza 12566, Egypt

**Keywords:** fexofenadine, ocular hydrogel, allergic conjunctivitis, Pluronic mixed micelles

## Abstract

This study was designed to formulate a polymeric mixed micelle (PMM) formulation to sustainably release fexofenadine (FEX) to treat allergic conjunctivitis effectively. A 3^2^ factorial design was employed where the studied factors were PL90G amount (X_1_) and Pluronic (F127 and P123) mixture ratio (X_2_), and the dependent variables were entrapment efficacy (EE, Y_1_, %), particle size (PS, Y_2_, nm), zeta potential (ZP, Y_3_, mV), and the percent of drug released after 6 h (Q6h, Y_4_, %). The optimized formula was blended with a hydrogel base to develop an FEX-PMM hydrogel, where the safety and efficiency of this hydrogel were evaluated using in vivo studies. The EE% of FEX-PMM ranged from 62.15 ± 2.75 to 90.25 ± 1.48%, the PS from 291.35 ± 6.43 to 467.95 ± 3.60 nm, the ZP from −5.41 ± 0.12 to −9.23 ± 0.23 mV, and the Q6h from 50.27 ± 1.11 to 95.38 ± 0.92%. The Draize test results confirmed the safety of the FEX-PMM hydrogel. Furthermore, the FEX-PMM hydrogel showed rapid recovery in animals with induced allergic conjunctivitis compared to the free drug hydrogel. These results assure PMM’s capability to deliver FEX to the conjunctival surface in a sustained pattern, consequently achieving better therapeutic outcomes.

## 1. Introduction

Allergic conjunctivitis is one of the most widespread external ocular diseases generated by abnormal immunohypersensitivity reactions to environmental allergens [1,2]. The immune pathophysiology involves activating immunoglobulin E (IgE)-mediated mast cells in conjunctival tissue, which releases preformed mediators, such as histamine, and triggers lipid-derived mediators and cytokines, leading to the widespread migration and infiltration of inflammatory cells to the ocular surface [3,4,5]. The biogenic amine histamine brings the initial clinical manifestations of allergic conjunctivitis [6]. Topical and oral antihistamines, mast cell stabilizers, and topical analgesics are the usual treatments for allergic conjunctivitis [7,8].

Fexofenadine (FEX) is a long-acting second-generation H1R antagonist that is non-anticholinergic and possesses anti-inflammatory effects. Due to its limited aqueous solubility (1.45 ± 0.15 mg/mL), FEX has poor oral bioavailability (33%) [9]. Several nanoformulations were designed to augment the oral bioavailability of FEX where Abdelhameed et al., 2022, formulated FEX-loaded nanostructured lipid carriers (NLCs) using a solvent injection approach and concluded that the ratio of solid lipid to liquid lipid and surfactant concentration impacted the NLC’s main characteristics, such as entrapment efficiency (EE%), particle size (PS), and zeta potential (ZP); also, the FEX-NLCs showed sustained release behavior over 48 h and enhanced oral bioavailability by 2.57 times compared to an FEX commercial product [10]. Furthermore, Sultan et al., 2022, used the homogenization technique to formulate FEX-loaded cubosomes where cubosomes with combined spherical and polygonal morphology sustained the FEX release over 8 h [11], while Nasr et al., 2020, succeeded in preparing FEX in spray-dried lactose-based enhanced in situ forming vesicles where the findings showed that utilizing surfactants with lower HLB values and longer alkyl chain length resulted in higher EE% of the hydrophobic drug (FEX), while the surfactants with higher HLB resulted in the faster dissolution of FEX [12]. Moreover, Abdelhameed et al., 2023, developed FEX nanosuspension fast-dissolving tablets where the dissolution rate of the nanosystem was significantly improved, with a subsequent 4-fold increase in the oral bioavailability of FEX [13]. Furthermore, FEX was formulated into intranasal microemulsions for treating allergic rhinitis, where the solubility of the drug was increased 14-fold compared to its intrinsic water solubility, while the in vitro drug release profile showed a biphasic release profile pattern [14]. Recently, El-Dakroury et al., 2024, developed chitosan-coated solid lipid nanoparticles for treating ulcerative colitis; the solid lipid nanoparticles were prepared using a hot homogenization technique followed by sonication where the EE% and PS of different formulae were affected by the molecular weight of chitosan, and the in vitro drug release study of the optimized formula showed a controlled release profile [15]. However, to date, no published study has attempted to develop a nanoformulation for ocular delivery of FEX to treat ocular allergy.

Conventional drug delivery systems used for topical treatment of allergic conjunctivitis do not always reduce itching discomfort or stop the destructive behavior that causes tissue damage [16,17]. Therefore, a more effective drug delivery system is required to release the drug to the ocular surface in a more controlled pattern.

Nanocarriers have substantial therapeutic value as drug delivery systems [18]. Nanocarriers are designed to adhere to the ocular surface, permitting greater drug permeation or deposition and prolonged drug release due to better tissue adherence [19].

Polymeric micelles are nanocarriers with core/shell structures of amphiphilic block copolymers consisting of a hydrophilic shell surrounding a hydrophobic core that self-assembles in water [20,21]. The hydrophilic shell is usually composed of polymers such as polyethyleneglycol (PEG), poly (ethylene oxide) (PEO), or poly (vinyl pyrrolidone). At the same time, the hydrophobic core is made up of polymers such as poly (propylene oxide) (PPO), poly (glycolic acid), and poly (D,l-lactic acid) [22].

Ocular polymeric micelle formulations have gained substantial interest due to their numerous exciting characteristics. Their capability to overcome ocular barriers and improve lipophilic drug delivery to the anterior and posterior eye segments has recently been highlighted [23]. Moreover, their exceptional core/shell structure allows the poorly soluble drugs to be encapsulated within the core of the micelle, increasing drug aqueous solubility and improving the permeation for hydrophobic medicines across the hydrophilic stroma [24]. Furthermore, polymeric micelle formulae might contain mucoadhesive polymers, such as Pluronic F127, which also demonstrated longer drug ocular residence time, leading to greater therapeutic efficacy [25].

Pluronics are amphiphilic block copolymers; under certain circumstances, Pluronics self-assemble into nanosized micelles where the hydrophilic PEO region is known as the corona, and mainly accommodates hydrophilic drugs, and the PPO region forms the core of the micelles in which poorly soluble drugs get solubilized [26].

These micelles have been successfully utilized to solubilize hydrophobic drugs such as naproxen, rofecoxib, camptothecin, tropicamide, haloperidol clozapine, and oxcarbazepine [27,28]. To enhance the thermodynamic stability of Pluronic micelles, phospholipids such as Phospholipon 90G (PL90G) can be incorporated into the Pluronic micelles to yield polymeric mixed micelles (PMMs). Due to the strong hydrophobic interactions between PL90G and the hydrophobic PPO blocks of the Pluronics, the thermodynamic stability of the micelles is raised [29,30].

No studies have been undertaken regarding preparing mixed micelles to incorporate FEX for treating allergic conjunctivitis. Therefore, FEX-loaded PMMs were prepared to sustain FEX release to the ocular surface. A 3^2^ factorial design was applied where the studied factors were PL90G amount (X_1_, mg) and Pluronic (F127 and P123) mixture ratio (X_2_), and dependent variables were entrapment efficacy (EE, Y_1_, %), particle size (PS, Y_2_, nm), zeta potential (ZP, Y_3_, mV), and percent drug released after 6 h (Q6h, Y_4_, %). The selected PMM formula was incorporated into the hydrogel base to develop an FEX-PMM-loaded hydrogel. The safety of the FEX-PMM hydrogel was assessed using the Draize test. Finally, the hydrogel’s efficacy was evaluated via a pharmacodynamic study where it was employed to treat rabbits with induced allergic conjunctivitis compared to free drug hydrogel.

## 2. Materials and Methods

### 2.1. Materials

Fexofenadine (FEX) was gifted by the European Egyptian Pharmaceutical Industry (Amreya, Alexandria, Egypt). Pluronic^®^ F127, Pluronic^®^ P123, and Spectra Por^©^ semi-permeable membrane tubing (12–14 kDa) were purchased from Sigma Chemicals Company (St. Louis, MO, USA). Phospholipon^®^ 90G (PL90G) was purchased from the Lipoid company (Ludwigshafen, Germany). Methanol, Polysorbate 80, and components of simulated lacrimal fluid were bought from El-Nasr Pharmaceutical Chemicals Co. (Cairo, Egypt). Hydroxy propyl methyl cellulose (HPMC K4m, 4000 cps) was obtained from Colorcon (Dartford, Kent, UK).

### 2.2. Experimental Design

Design Expert^®^ software (ver. 13; Stat-Ease Inc., Minneapolis, MN, USA) was used to create the runs of 3^2^ factorial design [31]. The studied factors were PL90G amount (X_1_, mg) and Pluronic (F127 and P123) mixture ratio (X_2_). The considered formulation variables are shown in Table 1. All FEX-PMM formulae contained Polysorbate 80 in a concentration of 20% of the PL90G content. The FEX-PMM formulae were evaluated by the measurement of the entrapment efficacy (EE, Y_1_, %), particle size (PS, Y_2_, nm), zeta potential (ZP, Y_3_, mV), and percent drug released after 6 h (Q6h, Y_4_, %).

### 2.3. Preparation of FEX-PMM

FEX-PMM formulae were made using the thin film hydration method followed by ultrasonication [32]. In a round-bottom flask, FEX (10 mg), PL90G, and Pluronics (F127 and P123 mixture in the required ratios) were dissolved in 10 mL methanol, then Polysorbate 80 (20% of PL90G content) was added to the solution. A rotary evaporator (Heidolph W 2000, Kehlheim, Germany), rotating at 150 rpm, evaporated the organic solvent at 60 °C under reduced pressure until a dry film was formed. The dry film was hydrated with 9 mL of distilled water, and the flask was rotated for 30 min under normal pressure at 60 °C to ensure complete hydration [33]. Then, the dispersion was sonicated for 60 s utilizing a probe sonicator (Newton, CT, USA). The prepared PMM formulation was kept at 4 °C until evaluation.

### 2.4. Characterization of FEX-PMM

#### 2.4.1. Drug Entrapment Efficiency

EE% was determined by centrifuging the samples at 14,000 rpm for 60 min at 2 °C using a cooling centrifuge (model 3K 30, Sigma, Osterode, Germany) to separate the unencapsulated precipitated FEX from the encapsulated PMM [34]. The supernatant was diluted with methanol and measured spectrophotometrically at 258 nm (UV-1900; Shimadzu Corp., Kyoto, Japan), where drug unloaded formulae were used as blanks during the spectrophotometric determination. The EE% was calculated using the following equation:

(1) $$\mathrm{EE}\%=\frac{Weight\;of\;drug\;in\;micelles}{Weight\;of\;added\;drug\;during\;preparation}\times 100$$

#### 2.4.2. Particle Size and Zeta Potential

Zetasizer (Zetasizer Nano ZS, Malvern Instruments, Worcestershire, UK) at 25 °C was used to estimate PS, polydispersity index (PDI), and ZP after the appropriate dilution (1:100) with deionized water [35].

#### 2.4.3. In Vitro Drug Release Study

The in vitro release of FEX from FEX-PMM formulae was performed using USP dissolution testing apparatus type II (model G2 Elite 8, Hanson Research Corp, Chatsworth CA, USA). The volume of dissolution media was 50 mL of simulated lacrimal fluid containing 0.05% sodium lauryl sulfate to ensure the sink condition [36,37]. The dissolution media were stirred at 25 rpm at 34 ± 0.5 °C. Three milliliters of the FEX-PMM formula (equivalent to 3 mg of FEX) was placed in an open-ended cylindrical glass tube covered with the Spectra Por semi-permeable membrane from one end and attached to the paddle of the apparatus from the other end [38]. One milliliter was withdrawn and substituted equally with fresh-release media at various points. A spectrophotometric analysis of the drug percentages released was performed at λ_max_ 258 nm [39]. The experiments were carried out in triplicates, and the mean (±SD) values were calculated for the released drug percentages and plotted against time. The results were fitted to various mathematical models to understand the possible release mechanism of FEX.

### 2.5. Selection of the Optimized FEX-PMM Formula

The optimized FEX-PMM formula was selected using the Design Expert^®^ software ver. 13. The desirability tool set the formula with the maximized Q6h, EE%, and minimized PS, while the ZP was chosen to be in the measured range of the formulae.

### 2.6. Characterization of the Optimized FEX-PMM Formula

#### 2.6.1. Morphology

The morphology of the selected FEX-PMM formula was identified by a transmission electron microscope (TEM) (JEM-1230, Joel, Tokyo, Japan). The dispersion was dropped on a carbon grid, stained by 1.5% phosphotungstic acid, and observed by TEM at 80 kV [40].

#### 2.6.2. Differential Scanning Calorimetry

The optimized formula was lyophilized in the presence of 5% *w*/*v* mannitol at −55 °C under vacuum using a freeze-drying system (Labconco, Kansas, MO, USA). Then, the thermodynamic analysis of FEX, Pluronic F127, Pluronic P123, PL90G, and lyophilized optimized FEX-PMM was established using differential scanning calorimetry (DSC-50, Shimadzu, Kyoto, Japan) in a temperature range of 25–300 °C at a heating rate of 10 °C/min [21].

#### 2.6.3. Stability Study

The optimal formula’s physical stability regarding drug leakage and physical changes was evaluated. The optimized formula samples were placed at 4 ± 1 °C for 90 days in glass vials tightly sealed with plastic closures. The EE%, PS, and ZP were measured and compared with the freshly prepared formula. Statistical significance was examined by Student’s *t*-test using Prism software (ver. 8, GraphPad, San Diego, CA, USA) [41].

### 2.7. Preparation of FEX-PMM Hydrogel

FEX-PMM-loaded hydrogel was prepared using 2% or 4% of HPMC k4M. The required weight of HPMC k4M was added to 1/3 of the necessary volume of deionized water at 80 °C to formulate the hydrogel base, and the blend was mixed for 15 min using a magnetic stirrer. The final volume was adjusted by either deionized water containing the selected FEX-PMM formula or deionized water having the same amount of FEX to prepare free drug hydrogel. The hydrogels were stored overnight at 4 °C [42].

### 2.8. Evaluation of FEX-PMM Hydrogel

#### 2.8.1. Determination of pH

The pH FEX-PMM-laden hydrogels were tested using a calibrated pH meter (Inolab pH 720, WTW, Weilheim, Germany) at 25 ± 1 °C.

#### 2.8.2. Rheological Studies

FEX-PMM-laden hydrogels’ viscosity was evaluated with a Brookfield digital DV-III Model viscometer (Middleboro, MA, USA) using spindle CP-52 and 25 rpm at 25 ± 1 °C to measure the viscosity.

#### 2.8.3. Evaluation of Gel Spreadability

To determine spreadability, a sample of 1 g of each FEX-PMM-laden hydrogel was applied between two glass slides and was compressed to uniform thickness by placing 20 g of weight on it. After 1 min, the weight was detached, and the diameter of the spread area (cm) was measured. Finally, the spreadability was calculated using the following equation [43].

Si = M × (L/T)(2)

Si is the spreading area, M is 20 g, L is the length of a glass slide (9.8 cm), and T is 1 min.

#### 2.8.4. Ex Vivo Mucoadhesive Strength

The mucoadhesive strength (MS) of the FEX-PMM-laden hydrogels was determined using a modified two-arm physical balance method [44]. In this method, one of the balancing arms—where the goat corneal mucosa was attached to the slides fronting the FEX-PMM-laden-hydrogel—was replaced with a base with a slide. After that, the hydrogel was placed on the slide and shielded by a second moving slide wired to the balance’s arm. Water droplets were added to the left pan to increase weight and put tension on the wire while the entire system remained motionless. The MS value was calculated based on the strength at which the hydrogel separated from either corneal mucosa surface [45]. The MS values of FEX-PMM-laden hydrogels were calculated using the following equation:

MS (dyne/cm^2^) = (m × g)/A (3)

where A is the area of the corneal mucosa in cm^2^, m is the mass of water in g, and g is the gravitational acceleration (981 cm/s^2^).

#### 2.8.5. In Vitro Drug Release Study

The in vitro release studies of FEX from the FEX-PMM-laden hydrogel were performed as mentioned in Section 2.4.3.

### 2.9. In Vivo Studies

#### 2.9.1. Animals

New Zealand Albino rabbits (2–2.5 kg) were obtained from Cairo University’s Faculty of Pharmacy, Giza, Egypt. The Research Ethics Committee of the Faculty of Pharmacy, Cairo University’s rules for the care and keeping of animals were followed. Also, the Research Ethics Committee approved the research protocol (Approval no. PI 1194).

#### 2.9.2. Draize Test

Six rabbits were classified into 2 groups of three to assess the degree of irritation. Before testing, both eyes in each animal were examined to ensure no defects. The lower lid was then gently pulled away from the eyeball to make a cup into which the right eye received the recommended dose (0.1 mL) of the FEX-PMM hydrogel (GP1) or free drug hydrogel (GP2). The left eye served as the control in both groups. The conjunctiva, eyelids, cornea, and iris were observed for any symptoms of irritation caused by the free drug hydrogel or FEX-PMM hydrogel [46]. After instillation, the ocular irritation score was assessed at 1, 2, 6, 8, 12, and 24 h using the following scale where the corneal opacity, iris swelling or congestion, and conjunctival redness or swelling were measured on a scale from 0 to 4, 0 to 2, and 0 to 3, respectively [47].

Following the Draize test, a histopathological examination was performed to detect the safety of the FEX-PMM hydrogel on ocular tissues. The rabbits were sacrificed using phenobarbital sodium (60 mg/kg) injection in the marginal vein [48]. Tissue conjunctiva specimens were rinsed in saline, sectioned, and then stained with Hematoxylin and Eosin (H&E) for histopathological examination. The slides were examined by an Olympus BX43 light microscope (Olympus, Hamburg, Germany) coupled with a camera linked to the Cellsens dimensions software ver 2.1 (Olympus, Tokyo, Japan). Also, the thickness of epithelium covering the conjunctiva was measured in the different groups using a Java-based image-processing program [49].

#### 2.9.3. Pharmacodynamics Study

Twenty-four rabbits were divided into four groups, each of six. The first group (GP1) was the negative control, where the healthy rabbits’ eyes received saline, while the rest of the animals received two drops of the histamine solution (1%) in both eyes. The eyes were observed until the maximum level of hyperemia was reached, which occurred 30 min after the instillation of the histamine solution [50]. The second group (GP2) was a positive control, where the inflamed rabbits’ eyes received no treatment. The right eye of each rabbit in the third group (GP3) was treated with free drug hydrogel (0.1 mg of FEX).

In comparison, in the fourth group (GP4) was FEX-PMM hydrogel (0.1 mg of FEX). Observations were made after applying the selected formulations at different time intervals. The rabbit’s conjunctiva in each group was examined and photographed. The histopathological examination and the thickness of epithelium covering the conjunctiva were measured in the different groups, as mentioned before in the Draize test.

### 2.10. Statistical Analysis of Data

The generated models of EE%, PS, and ZP were obtained using Design Expert ver. 13. The results were analyzed using ANOVA to determine the significance of the obtained models. Differences were regarded as significant at *p* < 0.05.

## 3. Results and Discussion

### 3.1. Factorial Design Optimization

During the primary studies to explore the variables that could impact the characteristics of PMM formulae, it was found that the amount of PL90G and Pluronic (F127 and P123) mixture ratio time influenced the PMM characteristics; therefore, they were set as independent factors as displayed in Table 2. The analysis of the design is illustrated in Table 3.

### 3.2. Characterization of FEX—PMM

#### 3.2.1. Drug Entrapment Efficiency

**Figure 1:**
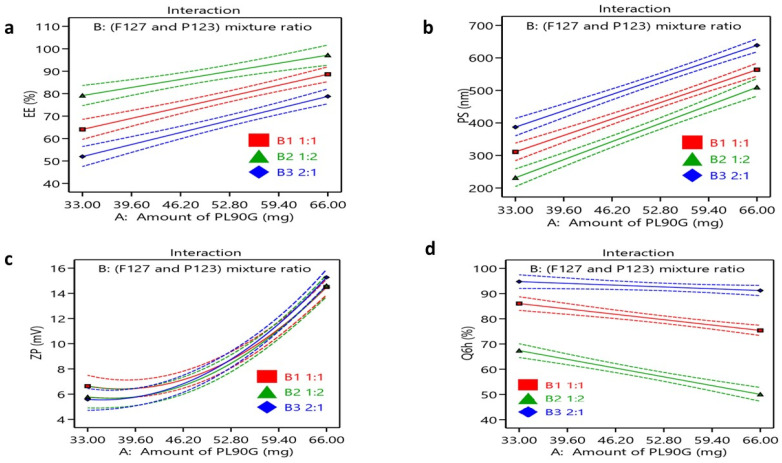
Interaction plots for the effect of the amount of PL90G (X_1_) and Pluronic (F127 and P123) mixture ratio (X_2_) on the (**a**) entrapment efficiency percentage, (**b**) particle size, (**c**) zeta potential, and (**d**) percentage of FEX released from the PMM formulations after 6 h.

High EE% of hydrophobic drugs in the polymeric micelles is desirable as encapsulating these drugs in micelles enhances their solubility, leading to better absorption and therapeutic outcomes [51]. The EE of FEX-PMM ranged from 51.20 ± 2.75 to 97.25 ± 1.48%, as shown in Figure 1a. The ANOVA analysis indicated that the amount of PL90G (X_1_) had a significant impact (*p* < 0.0001) on the EE% of FEX in the PMM formulations, as well as the Pluronic (F127 and P123) mixture ratio (X_2_) (*p* < 0.0001). Earlier studies have exposed that poorly soluble drugs can be loaded into polymeric micelles through hydrophobic interactions, hydrogen bonding, and van der Waals forces [52]. This might illuminate the increased EE% of FEX in F9 (90.25 ± 1.48%), which contained the highest amount of PL90G and P123. Increasing the PL90 G concentration was associated with a significant increase in the EE% as the addition of phospholipids to polymeric micelles increases the hydrophobicity of the PMM core, allowing for the solubilization of higher drug amounts. Moreover, it was observed that increasing the PL90G content reduces the possibility of drug leakage in the external phase as it forms multilayers around the particle. The Pluronic (F127 and P123) mixture ratio results showed that increasing the P123 percentage significantly increased EE% (*p* < 0.0001). Although both Pluronic F127 and P123 are composed of PEO-PPO-PEO with block lengths of 100-65-100 and 20-69-20, respectively, P123 possesses a higher PPO/PEO ratio.

Moreover, the hydrophilic–hydrophobic balance value (HLB) of P123 is lower than that of F127, 8 and 22, respectively [53]. Therefore, P123 is relatively hydrophobic compared to F127, providing a higher solubilization of the hydrophobic drug (FEX) through hydrophobic interactions [54]. These results revealed that the compatibility between FEX and the core of the PMM was better in the presence of a high P123 percentage. These findings agree with Fares et al., 2018, who found that increasing the P123 percentage has increased the EE% of hydrophobic drugs due to its better solubilization efficiency than F127 [55].

The coded equation was EE% = +76.63 + 11.56 ×A−0.27 × B [1] + 11.53 × B [2] + 0.71 × AB [1]−2.56 × AB [2].

#### 3.2.2. Particle Size

PS is one of the most critical features of PMM, affecting their physical properties and their in vivo performance as drug delivery carriers [56,57]. The nanoscale of polymeric micelles has great potential, as these nanomicelles provide a large surface area for efficient intraocular absorption with minimal eye irritation and tear production [58]. The optimum size for ocular delivery usually ranges from 30 to 300 nm [59]. Therefore, to obtain FEX-PMM with optimum size, the PMM formulae were prepared at varied polymeric ratios, and the size and distributions were studied. The mean PS of all PMM formulations was determined and ranged from 236.35 ± 6.43 to 633.50 ± 2.44 nm. The results showed that both X_1_ and X_2_ significantly influenced the PS of the PMM, as shown in Figure 1b. The amount of PL90G (X_1_) significantly affected the PS of the PMM formulae (*p* ˂ 0.0001). Additionally, the results indicated that the Pluronic (F127 and P123) mixture ratio (X_2_) had a significant impact on the PS of the FEX-PMM (*p* < 0.0001). The increase in the Pluronic F127 percentage within the ratio resulted in larger PS, while increasing the P123 percentage resulted in smaller PS. These results agree with the EE% outcomes, in which increasing PL90G resulted in a higher EE% of FEX, which led to increased PS where the Pluronic PPO chains and PL90G alkyl chains are assembled into a core of mixed micelles surrounding the hydrophobic core that is a shell comprised of PL90G polar head groups and PEO of Pluronics, while the lipophilic drug FEX was encapsulated in the lipophilic core of the mixed micelles. The increase in the Pluronic F127 percentage within the ratio results in larger PS, while increasing the P123 percentage results in smaller PS, where F127 has a larger molecular weight and a high percentage of PEO compared to P123 (12,600 g.mol^−1^ versus 5750 g.mol^−1^), so decreasing its amount led to PS reduction [60]. Also, at the same length of the PPO segment of P123 and F127, a longer PEO chain of F127 led to more hydrophilic properties of F127, thus enlarging the micellar size. These results agree with previous studies that concluded that the PPO/PEO polymer ratio affected the micelle size [61,62]. The coded equation was PS = +440.22 + 130.30 × A−2.91 × B [1]−69.72 × B [2]−4.03 × AB [1] + 8.65 × AB [2].

The PDI of all FEX-PMM formulations showed a wide range of PDI values from 0.26 ± 0.005 to 0.57 ± 0.010.

#### 3.2.3. Zeta Potential

The ZP values are a significant indicator of the stability of nanocarriers [63]. The surface charge has a relevant impact on micelles’ in vivo behavior, where positively charged nanocarriers are known for being mucoadhesive via electrostatic interaction with negatively charged sialic acid residues present in the mucous of the cornea and conjunctiva, enhancing drug ocular retention. On the contrary, neutral or negatively charged nanocarriers possess greater mucus-penetrating properties [31,40]. The ZP values of the prepared FEX-PMM formulations varied from −5.52 ± 0.12 to −15.47 ± 0.33mV. The PLG90 amount (X_1_) significantly affected ZP, as shown in Figure 1c. As the lipid percentage increased, the ZP also increased, where the negative charge of the phosphate groups of PLG90 contributed to the negative surface charge of the micelles [64]. Although the Pluronic (F127 and P123) mixture ratio (X_2_) did not statistically significantly affect ZP values, some studies have pointed out that the presence of the non-ionic surfactant molecules led to the shielding surface charge of the PL90G, resulting in a reduction in the ZP values [65]. Despite the low values of the ZP, it was assumed that the presence of non-ionic polymers was adequate for stabilizing the PMM, as the PEO sequences sterically stabilize the micelles [66]. Furthermore, the incorporation of PL90G would increase the thermodynamic stability of the PMM due to the tight hydrophobic interactions with hydrophobic PPO blocks [67]. The coded equation was ZP = + = 7.65 + 4.40 × A + 0.171 × B [1] −0.201 × B [2] −0.462 × AB [1] + 0.017 × AB [2] + 2.75 [A^2^].

#### 3.2.4. In Vitro Drug Release Study

**Figure 2:**
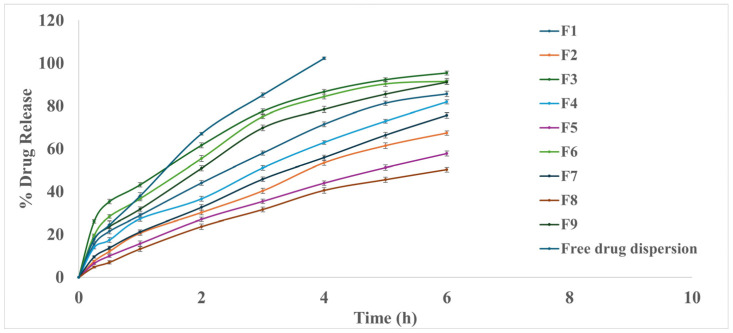
In vitro release profiles of FEX from different PMM formulae and drug aqueous dispersion.

Polymeric micelles can be designed to provide controlled and sustained release of encapsulated drugs. The release profile can be influenced by factors such as the type of polymer used, molecular properties of drug and drug–core interactions, polymer/surfactant used, preparation method, and micelle’s structural characteristics [68]. Enhancing the hydrophobicity and rigidity of the micellar core by incorporating PL90G could limit the movement of water and free ions to the micellar core, leading to more sustained release [69]. The ANOVA showed that both factors (X_1_ and X_2_) statistically affect Q6h%, as shown in Figure 1d. The Q6h% ranged from 50.27 ± 1.11 to 95.38 ± 0.92%, as illustrated in Figure 2. All PMM formulae showed prolonged release compared to drug dispersion, which reached nearly 100% after 4 h. During the same time (after 4 h), the percent of FEX release from the PMM ranged from 40.54 ± 1.3 to 86.61 ± 1.0%, slower than drug dispersion. Also, the study revealed that an increase in the PL90G (X_1_) in the PMM formulations led to a slower drug release, while increasing the percentage of the Pluronic F127-to-P123 ratio resulted in a higher drug Q6h%. The locus of the solubilized drug within the PMM has a substantial impact on drug release; if the drug is present in the micellar core, it will release slowly compared to if it is present in the corona where most of FEX was solubilized in the PPO core region; therefore, the PEO-forming corona regions act as a barrier against the drug release [28], and that resulted in a slow and more sustained release of the drug. These results comply with Terreni et al., 2020, who concluded that the better the affinity and compatibility between a drug and polymer, the higher the loading content and the slower the drug release [70]. Also, increasing the percentage of the Pluronic F127-to-P123 ratio resulted in a higher drug Q6h%. This could be attributed to the hydrophilicity of F127, which enhances water penetration into the core of the micelles, forming hydrophilic channels. As a result of the interaction between the Pluronic copolymers’ hydrogen bonds and PEO chains, the PEO top of polymeric micelles may become more extended, leading to the formation of additional hydrophilic channels in the PEO shell, which enhances the drug diffusion from the PPO core of the micelles [34]. The in vitro release profile of FEX from PMM formulations was found to comply with the first-order release model, except for the formulations containing a 1:2 ratio of Pluronic F127 to P123, which followed the Higuchi release model. The coded equation was Q6h% = 77.45−5.25 × A + 3.24 × B [1] − 18.74 × B [2] − 0.068 × AB [1] − 3.4 × AB [2].

### 3.3. Selection of the Optimized FX-PMM Formula

The required constraints were to maximize EE% and Q6h% and minimize the PS, while the ZP was chosen to be in the measured range of the formulae, |5.52−15.47|. The software with a desirability value of 0.936 suggested the FEX-PPM formula (F3).

### 3.4. Characterization of the Optimized FEX-PMM Formula

#### 3.4.1. Morphology

**Figure 3:**
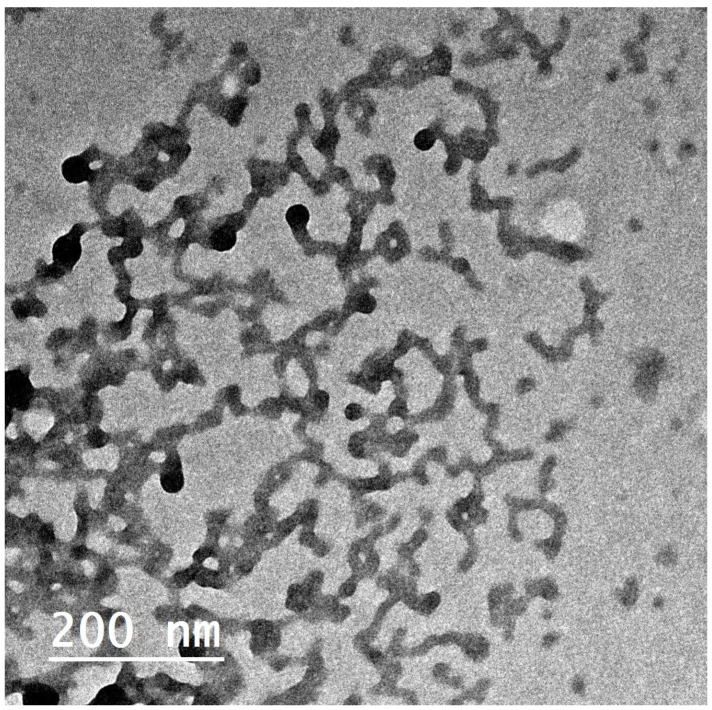
TEM micrographs of the selected PMM formula (F3).

The TEM micrographs of the optimized FEX-PPM formula (F3) in Figure 3 show nanosized tubular polymeric micelles besides spherical ones. The TEM micrographs indicated a less homogenous dispersion due to different morphologies (tubular and spherical micelles). This explained the wide range of PDI values. These results comply with Basalious et al., who found that increasing the hydrophobicity of PMM through the addition of phospholipids and decreasing the proportion of Pluronics F127 thus enhanced the transformation of micelles from a spherical shape to a tubular one [29]. Also, increasing the ratio of the hydrophobic Pluronic P123, which usually forms cylindrical aggregates in the aqueous medium rather than the spherical micelles formed by a hydrophilic Pluronic F127, and these cylindrical aggregates is found to exhibit a higher solubilization capacity than spherical micelles [71].

#### 3.4.2. Differential Scanning Calorimetry Studies

**Figure 4:**
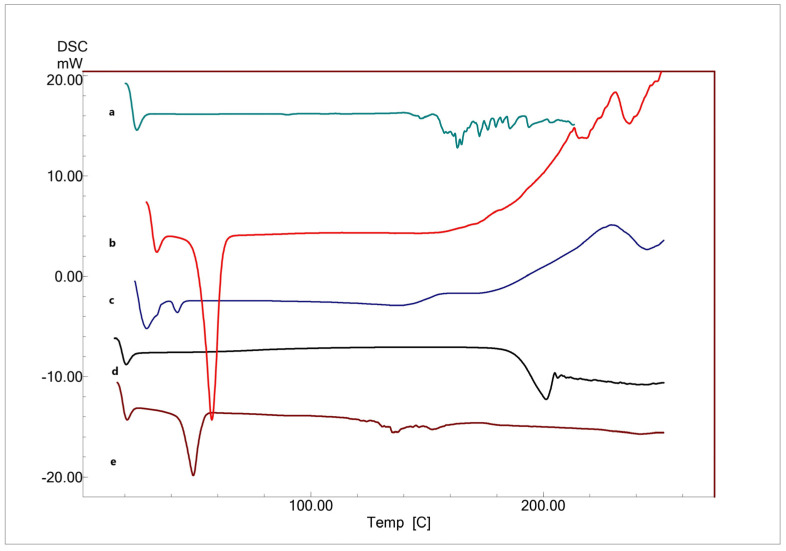
DSC thermograms of (a) PL90 G, (b) Pluronic F127, (c) Pluronic P 123, (d) FEX, (e) lyophilized optimized formula.

DSC was performed for the plain powder of FEX, Pluronic F127, Pluronic P123, PL90G, and FEX-PMM to assess the phase transformation of FEX through the formation of tubular micelles. As shown in Figure 4, the DSC thermogram for FEX revealed a distinctive single, sharp endothermic peak at 201.15 °C corresponding to FEX’s melting point, confirming its crystallinity. The thermogram of PL90G showed multiple endothermic peaks at 163.09, 172.5, 176.16, 185.69, 193.75, and 203.17 °C. The thermogram of Pluronic F127 showed an endothermic peak at 57.41 °C, while Pluronic P123 peaked at 41.34 °C. The DSC thermogram of FEX-PMM showed a complete disappearance of the drug peak and shifting of PL90G’s endothermic peaks to lower temperature and intensity (146.69 and 152.31 °C). The disappearance of the endothermic peak of FEX in the DSC thermogram of FEX-PMM indicated that FEX was solubilized in the hydrophobic core of the PMM [72]. The transformation of FEX from crystalline to amorphous or molecularly dispersed form is beneficial for enhancing the dissolution as an amorphous form of the drug does not require energy to break up the crystalline lattice [73]. This enhancement in the solubility and dissolution rate of FEX contributed to improved absorption in the ocular environment; therefore, the desired therapeutic concentration could be achieved [74].

Additionally, the shifting of PL90G’s endothermic peaks to lower temperature and intensity could be attributed to the fact that the phase transition temperature of the colloidal dispersion was consistently lower than that of the anhydrous lipid.

#### 3.4.3. Stability Study

After storing the optimized formula at 4 °C for 90 days, the EE%, PS, and ZP were evaluated. The paired *t*-test, *p* > 0.05, indicated an insignificant deviation from the freshly prepared formula: the EE% measurement was 80.96 ± 1.3%, and the ZP value was −5.67 ± 0.3 mV. However, there was a slight increase in the PS of the PMM formula, 251.81 ± 3.76 nm, compared to the fresh formula PS, 236.35 ± 6.43 nm, 43 nm, which might be due to the slight aggregation of hydrophobic micelle cores during the storage period [75].

### 3.5. Evaluation of FEX-PMM Hydrogel

#### 3.5.1. Determination of pH

The pH of the gels was compatible with the pH of tears (7.4), as shown in Table 4. Thus, it may be concluded that these systems are tolerable and would not be anticipated to irritate the eyes’ surface [76].

#### 3.5.2. Rheological Studies

The FEX-PMM hydrogels displayed a thixotropic pseudoplastic flow behavior, presenting an immediate flow after stress application. The thixotropic property offers excellent potential in ocular drug delivery because the hydrogels can transform into the sol state under the shear induced by physical blinking [77].

#### 3.5.3. Measurement of Gel Spreadability

Spreadability is defined as the size of the area to which it effortlessly spreads after application to the target site. Ocular gels that spread more within less spreading time are considered optimal as these formulations ensure the uniform distribution of the drug across the ocular surface, which is necessary for effective ocular absorption [78]. The spreadability values for FEX-PMM-loaded ocular hydrogels ranged from 7.26 ± 0.08 to 9.79 ± 0.23 (g.cm/s), indicating that they will spread readily at low shear stress. Since viscosity and spreadability are inversely correlated, increasing HPMC concentration significantly reduced the spreadability values. The spreadability values were dependent on the concentration of HPMC [36].

#### 3.5.4. Ex Vivo Muco-Adhesive Strength

The mucoadhesion approach can be advantageous in improving the ocular bioavailability of drugs by increasing their residence time on the ocular surface [79].

The concentration of HPMC significantly affected the MS of the prepared FEX-PMM-loaded ocular hydrogel gels, as increasing the HPMC concentration would increase the number of penetrating polymer chains per unit volume of mucin. Therefore, the MS value increased from 16,190 ± 62.09 to 18,147 ± 49.12 dyne/cm^2^ upon increasing HPMC concentration from 2 to 4%. The high MS values of FEX-PMM-loaded hydrogels suggested that these formulations had sufficient mucoadhesive strength to retain the drug for extended periods on the conjunctival surface, leading to improved drug absorption.

#### 3.5.5. In Vitro Drug Release Study

**Figure 5:**
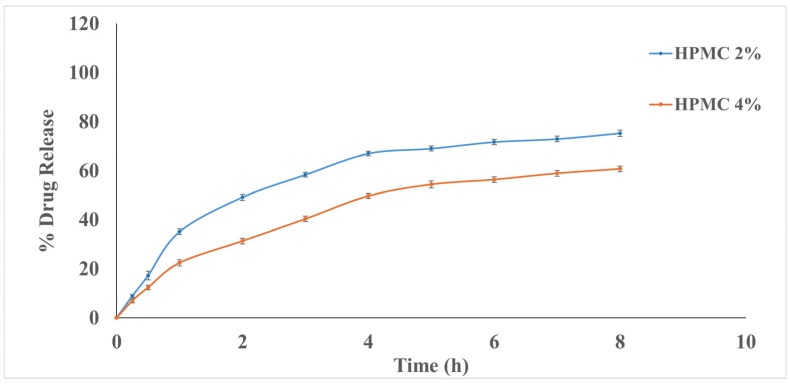
In vitro release profiles of FEX from FEX-PMM-loaded hydrogel.

Incorporating the FEX-PMM into 2% and 4% HPMC gel bases prolonged the drug release, where the Q8h was 75.15 ± 1.3 and 60.97 ± 1.20%, respectively, as illustrated in Figure 5. The sustained release may be due to the diffusional resistance of the gel in a semisolid matrix and the adhesion properties of the polymeric gel structure. Moreover, the diffusion boundary layer’s thickness limits drug molecules’ release into the aqueous buffer [80]. It is essential to mention that this prolonged release may benefit the treatment of allergic conjunctivitis due to continuous drug release. This controlled and sustained release is critical for maintaining therapeutic drug levels over extended periods, which is particularly beneficial in ocular applications where frequent dosing is often required [81]. The in vitro release profile of FEX from PMM-loaded hydrogels was found to comply with the Higuchi release model.

The hydrogel fabricated with 2% HPMC was chosen to be assessed through the in vivo studies due to its low viscosity and accepted Q8h%.

### 3.6. In Vivo Studies

#### 3.6.1. Draize Test

**Figure 6:**
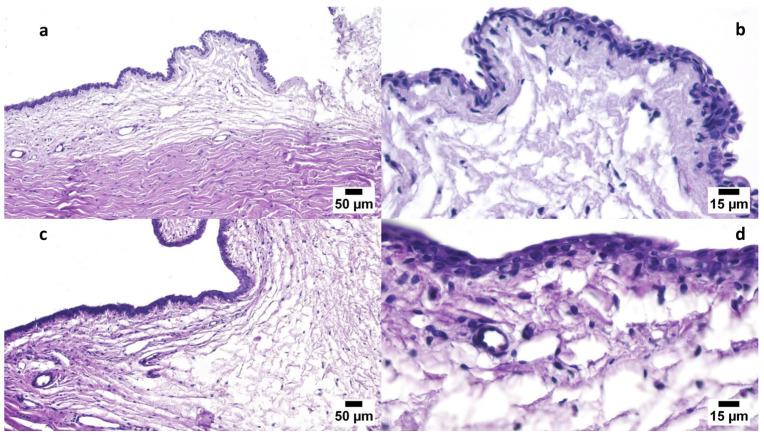
(**a**) A photomicrograph of GP1 conjunctiva, showing normal conjunctiva at a scale bar of 50 µm (H&E). (**b**) A photomicrograph of the GP1 conjunctiva, at higher magnification (15 µm), showing normal epithelial lining and lamina propria of the conjunctiva (H&E). (**c**) A photomicrograph of GP2 conjunctiva, showing normal conjunctiva at a scale bar of 50 µm (H&E). (**d**) A photomicrograph of the GP2 conjunctiva, at higher magnification (15 µm), showing normal epithelial lining and lamina propria of the conjunctiva (H&E).

**Figure 7:**
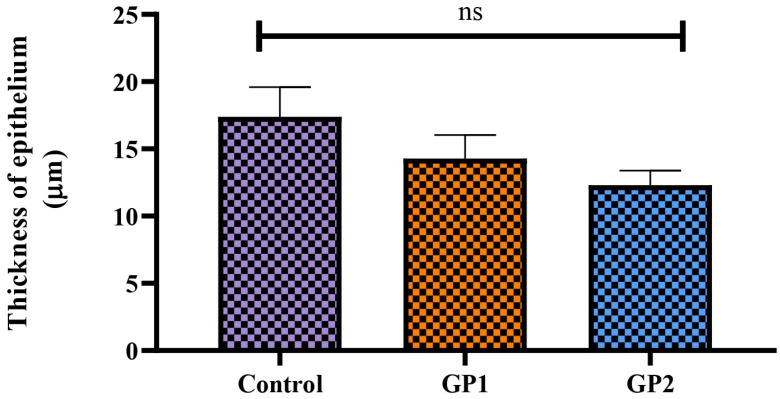
The thickness of the epithelium covering of conjunctiva in different groups is presented as means ± SD; ns, not significant.

This study assessed whether ocular irritation or damage could be caused by FEX-PMM hydrogel. This study compared the FEX-PMM hydrogel to a 2% HPMC gel base containing the same amount of FEX. There were no signs of irritation, redness, lacrimation, or congestion in either group. These findings were confirmed by the outcomes of histopathological examination, as shown in Figure 6a–d, which revealed a normal conjunctiva in all groups. Furthermore, upon analyzing the variation of epithelium thickness of the conjunctiva, there was no significant difference between the average normal thickness (left eye) and the tested groups, as shown in Figure 7. The *p* values of control versus GP1, control versus GP2, and GP1 versus GP2 were 0.4314, 0.1192, and 0.7088, respectively. Therefore, these results indicated that FEX-PMM hydrogel was non-irritating to the eyes.

#### 3.6.2. Pharmacodynamics Study

**Figure 8:**
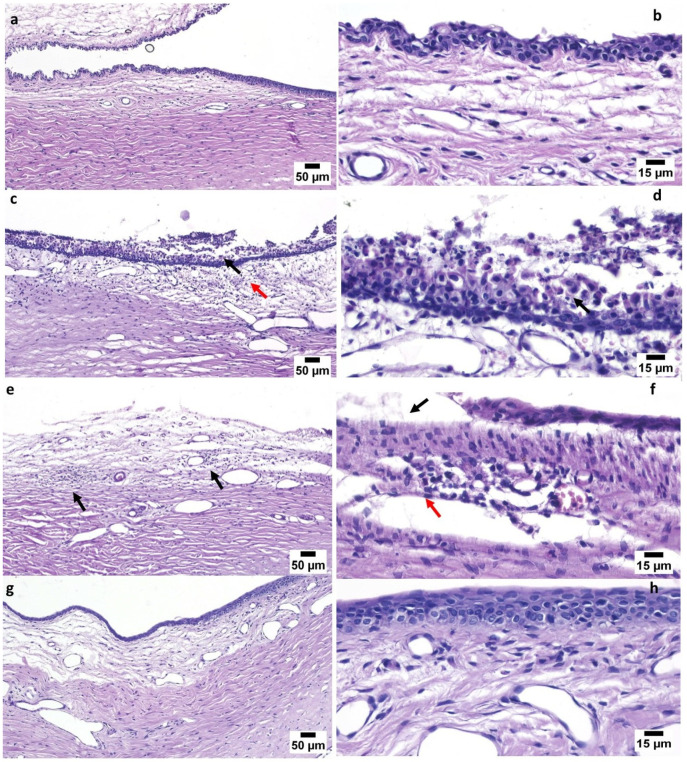
(**a**) A photomicrograph of the GP1 conjunctiva, showing normal conjunctiva structure (H&E). (**b**) A photomicrograph of GP1 conjunctiva at higher magnification, showing normal structure of stratified squamous epithelial lining and connective tissue of propria. (**c**) A photomicrograph of GP2 conjunctiva, showing intense inflammatory cell infiltration (red arrow) and sloughing of the epithelial lining (black arrow) of the conjunctiva. (**d**) A photomicrograph of GP2 conjunctiva, at higher magnification, showing intense inflammatory cell infiltration and desquamation of the epithelial lining (black arrow) of conjunctiva. (**e**) A photomicrograph of GP3 conjunctiva, showing mild edema and mononuclear inflammatory cell infiltration (arrow). (**f**) A photomicrograph of GP3 conjunctiva, at higher magnification, showing some sloughed areas in the lining epithelium (black arrows) and mild mononuclear inflammatory cell infiltration (red arrow). (**g**) A photomicrograph of GP4 conjunctiva, showing normal conjunctiva. (**h**) A photomicrograph of GP4 conjunctiva, at higher magnification, showing normal epithelial lining and lamina propria of the conjunctiva (H&E).

This study investigated the potential of the developed FEX-PMM hydrogel in treating rabbits with induced allergic conjunctivitis. The microscopic examination of the conjunctiva from the control group (GP1) revealed a normal histological structure of the conjunctival lining epithelium resting on connective tissue lamina propria, Figure 8a,b. The positive control group (GP2) showed intense edema with mononuclear and eosinophilic cell infiltration. The lining epithelium was also necrosed and sloughed; some regions within the epithelial surface were thickened (Figure 8c,d). Regarding the group treated with free drug gel (GP3), the examined sections revealed moderate improvement. The epithelial loss was focal and associated with mild mononuclear inflammatory cell infiltration and numerous congested blood vessels, Figure 8e,f. Marked improvement was noticed in the group treated with FEX-PMM hydrogel (GP4), as the examined sections were normal, Figure 8g,h.

**Figure 9:**
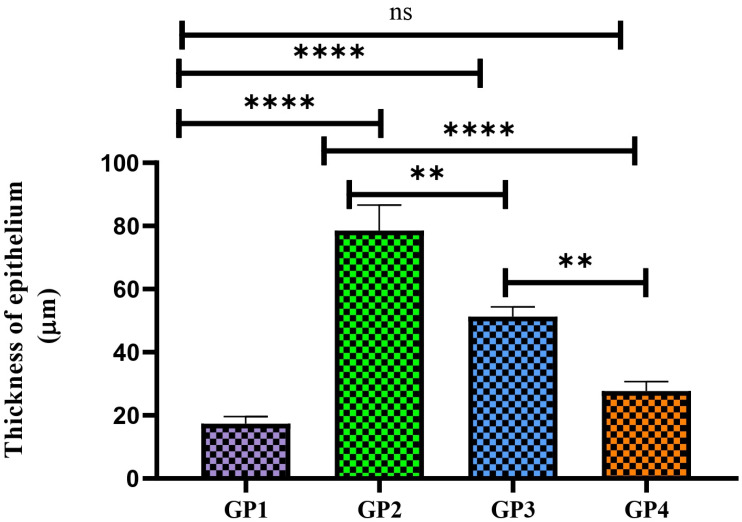
The thickness of the epithelium covering of conjunctiva in different groups is presented as means ± SD; ns, not significant; **** *p* < 0.0001; ** *p* < 0.01.

Moreover, the thickness of epithelium covering the conjunctiva was measured in different groups. A marked significant increase was detected in the positive group (GP2) compared to other groups. As illustrated in Figure 9, there is no significant difference between the negative control group (GP1) and the group treated with FEX-PMM hydrogel (GP4), indicating the ability of the developed formula to treat the inflamed epithelium. On the contrary, there was a significant difference between the group treated with free drug gel (GP3) and the negative control group (GP1), indicating incomplete recovery of the inflamed conjunctival epithelium.

The in vivo study results revealed the efficacy of the FEX-PMM hydrogel in treating allergic conjunctivitis, where it restored the normal conjunctival mucosa with normal thickness and normal lamina propria, while there was only a partial improvement in the group treated with the free drug gel. This enhancement might be due to the better solubilization of the drug in the PMM compared with the free drug gel. Additionally, the capability of Pluronic F127 and HPMC to enhance the mucoadhesion of the FEX-PMM hydrogel resulted in prolonged residence time on the ocular tissues, achieving higher therapeutic efficacy [82]. These results agree with Pescina et al., 2019, who studied the ex vivo conjunctival retention and transconjunctival penetration of hydrophobic drugs using polymeric micelles and concluded that polymeric micelles are very useful to solubilize hydrophobic drugs, enhancing the ocular retention time and the penetration across epithelia of the conjunctiva [83].

## 4. Conclusions

This study aimed to design polymeric mixed micelles composed of Pluronic P123 and F127 loaded with the poorly soluble antihistaminic drug FEX. The optimization of FEX-loaded P123/F127 mixed micelles was carried out by a 3^2^ design. The amount of phospholipids and the mixture ratio of Pluronics impacted the characteristics of the prepared formulae. The optimized formula showed high entrapment efficiency and minimized particle size. The FEX-PMM-loaded hydrogel showed sustained release due to the drug’s encapsulation into the micelles’ inner core. There were no signs of ocular damage or irritation in the rabbits’ eyes after administering the FEX-PMM hydrogel. Furthermore, the FEX-PMM hydrogel induced the complete restoration of the normal histology of the conjunctival mucosa. Conclusively, polymeric mixed micelles provided a promising nano-delivery system for the ocular delivery of poorly soluble drugs.

## Figures and Tables

**Table 1:** The 3^2^ factorial design for the optimization of FEX-PMM Formulae.

Factors	Levels		
X_1:_ PL90G amount (mg)	33	49.5	66
X_2_: Pluronic (F127 and P123) mixture ratio	1:1	1:2	2:1
Response	Desirability constraints		
Y_1_: EE (%)	Maximize		
Y_2_: PS (nm)	Minimize		
Y_3_: ZP (mV)	In the measured range of formulae		
Y_4_: Q6h (%)	Maximize		

PL90G, Phospholipon 90G; EE, Entrapment efficiency; PS, Particle size; PDI, Polydispersity index; ZP, Zeta potential; and Q6h%, Percent drug released after 6 h.

**Table 2:** Experimental runs, and independent and dependent variables of FEX—PMM following a 3^2^ factorial design.

Formula Code	PL90G Amount (mg) (X_1_)	Pluronic (F127 and P123) Mixture Ratio (X_2_)	EE % (Y_1_)	PS (nm) (Y_2_)	PDI	ZP (mV) (Y_3_)	Q6h (%) (Y_4_)
F1	33	2:1	51.20 ± 2.75	385.95 ± 4.23	0.33 ± 0.008	−5.52 ± 0.12	85.60 ± 1.26
F2	33	1:1	62.70 ± 1.41	302.20 ± 9.03	0.45 ± 0.002	−6.67 ± 0.36	67.33 ± 1.03
F3	33	1:2	79.35 ± 2.61	236.35 ± 6.43	0.49 ± 0.005	−5.84 ± 0.12	95.38 ± 0.92
F4	49.5	2:1	66.99 ± 2.19	515.95 ± 3.60	0.48 ± 0.025	−7.76 ± 0.05	81.84 ± 0.98
F5	49.5	1:1	79.10 ± 1.62	455.45 ± 5.41	0.26 ± 0.005	−7.82 ± 0.48	57.74 ± 1.17
F6	49.5	1:2	87.87 ± 3.53	360.95 ± 5.16	0.41 ± 0.043	−7.32 ± 0.28	91.46 ± 1.38
F7	66	2:1	77.80 ± 0.92	633.50 ± 2.44	0.57 ± 0.010	−15.47 ± 0.33	75.57 ± 1.29
F8	66	1:1	89.48 ± 1.90	559.05 ± 1.62	0.52 ± 0.020	−14.12 ± 0.53	50.27 ± 1.11
F9	66	1:2	97.25 ± 1.48	514.00 ± 6.26	0.47 ± 0.018	−14.67 ± 0.23	91.07 ± 1.06

Abbreviations: PL90G, Phospholipon 90G; EE, Entrapment efficiency; PS, Particle size; PDI, Polydispersity index; ZP, Zeta potential; and Q6h%, Percent drug released after 6 h.

**Table 3:** The results of the 3^2^ factorial design analysis.

Source	EE (%)	PS (nm)	ZP (mV)	Q6h (%)
*p* Value	<0.0001	<0.0001	<0.0001	<0.0001
Model	Linear	Linear	Quadratic	2F1
X_1_ = A; Amount of PL90G	<0.0001	<0.0001	<0.0001	<0.0001
X_2_ = B; Pluronic (F127 and P123) mixture ratio	<0.0001	<0.0001	0.4646	<0.0001
Adequate precision	31.96	31.96	36.44	52.00
R^2^	0.9895	0.9895	0.9977	0.9969
Adjusted R^2^	0.9791	0.9791	0.9939	0.9939
Predicted R^2^	0.9345	0.9345	0.9865	0.9654
Significant factors	X_1_, X_2_	X_1_, X_2_	X_1_	X_1_, X_2_

Abbreviations: PL90G, Phospholipon 90G; EE, Entrapment efficiency; PS, Particle size; PDI, Polydispersity index; ZP, Zeta potential; and Q6h%, Percent drug released after 6 h.

**Table 4:** The composition and physical characteristics of different FEX-PMM-laden ocular hydrogels.

HPMC Percent (%)	pH	Spreadability (g.cm/s)	MS (dyne/cm^2^)	Viscosity Cp	Qh8 (%)
2%	7.4 ± 0.15	9.79 ± 0.23	16,190 ± 62.09	698.5 ± 20.50	75.15 ± 1.3
4%	7.3 ± 0.2	7.26 ± 0.08	18,147 ± 49.12	2397 ± 20.75	60.97 ± 1.20

## Data Availability

Data are contained within the article.
